# Interest of Lung Ultrasound in the Management of Acute Heart Failure in Post-Emergency Service

**DOI:** 10.3390/life15050752

**Published:** 2025-05-07

**Authors:** E. Bel Alonso, A. Grember, C. Cheval, R. Papillon, L. Mairot, A. Deroux, L. Bouillet, A. Bellier, P. Dumanoir

**Affiliations:** 1 Emergency Department and Mobile Intensive Care Unit, University Hospital of Grenoble Alpes, 38700 La Tronche, France; 2 Emergency Department and Mobile Intensive Care Unit, University Hospital of Besançon, 25030 Besançon, France; 3 Department of Internal Medicine, University Hospital of Grenoble Alpes, 38700 La Tronche, Francepdumanoir@chu-grenoble.fr (P.D.); 4 CNRS TIMC Laboratory, UMR 5525, University of Grenoble Alpes, 38700 La Tronche, France

**Keywords:** acute heart failure, pulmonary congestion, lung ultrasound

## Abstract

Lung ultrasound (LUS) has emerged as a simple, rapid, and non-invasive method for the dynamic assessment of pulmonary congestion, a major prognostic factor and a therapeutic target in acute heart failure (AHF). In a single-center prospective observational study, 42 patients hospitalized for AHF in the post-emergency polyvalent medicine department of CHU Grenoble were successively included between May 2021 and July 2022. Patients undergoing hemodialysis, those with pneumonectomy or lung fibrosis, or those placed under guardianship or deprived of freedom were excluded. Clinical examination, LUS, and electrolyte panel results were collected daily. Vital status was assessed 30 days after the last LUS. The primary endpoint was the evolution of the number of B-lines in relation to the dose of diuretic administered. Secondary endpoints included the evolution of B-lines according to clinical signs of congestion and plasma creatinine levels, the agreement between LUS and clinical findings at discharge, and the prognostic value of LUS at discharge for 30-day re-admission for AHF and all-cause mortality. A total of 188 LUS were performed. The patients were elderly (85.8 years [SD 8.1]) and comorbid. The median number of B-lines decreased from 17 at admission to 7 mid-hospitalization, then stabilized. The median daily intravenous diuretic dose declined from 40 mg to 20 mg. Patients with chronic kidney disease (CKD) had more B-lines at admission (24.2 (SD 11.6) vs. 8.2 (SD 8.8)). However, B-line evolution was independent of creatinine levels. Higher B-lines at discharge were significantly associated with 30-day mortality (15.2 vs. 3.9, *p* < 0.001). In the absence of a gold standard for the assessment of pulmonary congestion, LUS appears to be an additional tool for optimizing the management of AHF.

## 1. Introduction

Heart failure is a major public health problem. In France, this frequent and growing pathology affects 2.3% of the adult population and is responsible for 70,000 deaths per year. Acute heart failure (AHF) is one of the leading causes of hospitalization in patients over 65 years old, with hospital stays often being long and expensive [1].

Pulmonary congestion appears as a major prognostic factor in AHF [2,3], and therefore represents an important therapeutic target [4]. However, current methods for assessing congestion in medical wards are imprecise and lack standardization. Clinical evaluations are often insensitive, even when performed by experienced physicians [5], and pulmonary auscultation suffers from poor reproducibility. Despite this, these subjective methods remain the primary basis for adjusting diuretic therapy. Studies are currently underway to standardize this care, such as the CARESS protocol within the PRODUCT HF study [6].

Lung ultrasound (LUS) is an easy-to-use, rapid, reproducible, and non-invasive method that offers bedside evaluation of pulmonary congestion status [7]. Minimal training is required, and reproductible results are obtained after 30 min to 1 h of practice [8]. A B-line, or comet tail, is an ultrasound artefact characterized by a vertical hyperechogenic line starting from the pleural line, not fading, and moving synchronously with lung sliding. It reflects the presence of engorged interlobular septa, areas where air is in close contact with water, leading to decreased air content and increased lung density due to differences in acoustic impedance. The number of B-lines increases with the reduction in air content. They are correlated with the presence of an alveolar-interstitial syndrome, with a sensitivity and a specificity of 93% [9,10].

The value of LUS for the diagnosis of AHF has already been proven, particularly in patients admitted to the emergency department (ED) for acute dyspnea [8,9,10]. Monitoring B-lines could be interesting for tracking pulmonary congestion, allowing for treatment in AHF patients and improving their prognosis. Diuretic administration is commonly followed by a rapid decrease in the number of B-lines [11].

The goal of this prospective observational study is to evaluate whether changes in B-lines observed on LUSs can reliably reflect pulmonary congestion status and correlate with diuretic dosing, as well as clinical and biological signs, in AHF patients hospitalized in a polyvalent medicine unit. This could support more precise, responsive, and standardized management of AHF in routine clinical care.

## 2. Materials and Methods

### 2.1. Study Design

We conducted this single-center prospective cohort study in the internal medicine department of Grenoble Alpes University Hospital (CHUGA). This department admits a large number of patients directly from the ED and with any type of medical pathologies.

The senior doctors working in this department are emergency physicians, general practitioners, geriatricians, and interns.

### 2.2. Population

Patients admitted to the internal medicine department from ED between 18 May 2021, and 15 July 2022, were screened for inclusion. Patients were eligible if they were over 18 years-old and presented an AHF at admission. The diagnosis was assessed by two senior physicians, according to the diagnostic criteria of the European Society of Cardiology (ESC) guidelines [12]. Patients undergoing hemodialysis, those with pneumonectomy or lung fibrosis, and those under guardianship or deprived of freedom were excluded.

### 2.3. Heart Failure Treatment and Protocol

Patients were treated for AHF according to usual practices and with the help of a protocol created by the cardiologists at CHUGA which summarizes the guidelines of the ESC (Appendix A). Patients with severity criteria (hypoperfusion, cardiogenic shock) were managed in the cardiology department. This protocol uses clinical assessment, weight, and diuresis to guide therapeutic adjustments. Ultrasound techniques are not available in the department. The protocol had been in use for several months before the start of the study and physicians were encouraged to follow it; however, adaptation of diuretic posology was left to their discretion.

### 2.4. Lung Ultrasound Protocol

For each patient, LUS is performed at admission and daily by a senior physician from the department who is not in charge of the patient and is blinded to clinical and biological findings.

A patient’s position can influence the number of B-lines [7,13]. Therefore, in this study, for each bedside LUS, we positioned our patients in a 30° supine position using adjustable beds. Inspired by the BLUE-protocol of Lichtenstein [10,14] and according to the expert consensus document [15], four points on each hemithorax were analyzed: two anterior points between the sternum and the anterior axillary line, i.e., one under the clavicle and one under the nipple, and two lateral points between the anterior and posterior axillary lines, i.e., one superior and one inferior.

For each area, the number of B-lines was counted, and the presence of pleural effusion was recorded. In areas where pleural effusion was present, the number of B lines was not counted. Results were reported on a paper form (see Appendix B) completed by the physician for each exam and kept sealed.

A GE Healthcare Vivid T8 echograph device with a 3.5–5 MHz phased array transducer at an imaging depth of four to six centimeters was used. The depth was set according to the department’s standard practices to ensure standardized image reading, i.e., it could be adjusted according to the patient’s size.

Most of the physicians already had echography certification, while others completed a theoretical and practical course before starting the inclusions.

### 2.5. Follow-Up/Data Collection

Medical information about the patient’s status was recorded at admission and updated daily by the physician responsible for the patient (see Appendix C) in a nominative data collection notebook (Appendix D).

Demographic variables (age, sex), past medical history and comorbidities (Modified Charlson score), usual treatments, heart failure characteristics (etiology, ejection fraction), physical examination findings, and clinical variables (height, weight, vital signs, heart failure signs and symptoms), as well as the results of electrocardiogram, chest radiography, and a biological test (hemogram, electrolyte panel and renal function, martial assessment, liver function test, natriuretic peptides, troponin, and nutritional status) were recorded at admission.

Every day, including on the day of discharge, clinical signs and symptoms of heart failure, vital parameters, administered diuretic treatment, and available electrolyte panel were recorded.

On the day of discharge, we collected information on the triggering factor of cardiac decompensation, whether iron was administered during hospitalization, nutritional and kinetic assessment, and planned follow-up (both biological and medical). Patients who remained in the hospital after the resolution of AFH for a social issue (institutionalization or implementation of home help) or secondary medical issue were considered discharged after 48 h of stable diuretic treatment.

Vital status and possible need for rehospitalization were assessed 30 days after the last LUS by reviewing hospital medical records or via phone calls to the patient, their family, or their attending physician.

### 2.6. Study Outcomes

The primary endpoint examined was the daily measurement of the number of B-lines in LUS compared to the evolution of diuretic doses.

Several secondary outcomes were assessed: the evolution of the number of B-lines according to clinical signs of cardiac overload (crackles and lower limbs oedemas) and the agreement between them at hospital discharge; the evolution of the number of B-lines according to plasma creatinine level; and the prognostic value of LUS at discharge in predicting readmission for AHF and all-cause mortality at 30 days.

We also aimed to assess the feasibility of daily monitoring of LUS during working days, as well as the interobserver reproducibility of LUS by two doctors on the same group of patients on the same day.

### 2.7. Statistical Analysis

We calculated that with a sample size of 42, a two-sided 95% confidence interval for a single mean would extend one from the observed mean, since the standard deviation was known to be 3.28 and the confidence interval was based on the large sample z statistic.

For the statistical analysis, the duration of follow-up for each patient was divided into four parts, with 100% representing the total time of hospitalization. A descriptive statistical analysis was performed on the baseline characteristics of the population. Continuous variables are reported as median (interquartile range (IQR)) or mean value (standard deviation (SD)), as appropriate. Categorical variables are expressed as percentage (%).

In the analysis of the primary endpoint, we first used a Join Point regression model describing B-lines and diuretic posology. Then, a temporal trend analysis was performed on the evolution of the number of B-lines and posology of diuretics. A linear regression model was employed to model the linear trend and to compare trends according to the hospitalization duration threshold defined as 0% (admission), 25%, 50%, 75%, or 100% (discharge). Adjustment for patient age was performed.

Assessments of the evolution of the number of B-lines according to clinical signs of cardiac overload, the relationship between B-lines and crackles at discharge, and the evolution of B-line count according to plasma creatinine level were performed using the same analysis as the primary endpoint.

The prognostic value of LUS at discharge for predicting readmission or all-cause mortality at 30 days was analyzed using a linear regression model.

For the reproducibility study, we calculated a correlation coefficient: an intraclass correlation coefficient for the quantitative variable (B-lines) and a Kappa coefficient for the qualitative variable (pleural effusion).

Statistical significance was set at *p* < 0.05. All analyses were performed using the programs RStudio (Version 2022.07.1, PBC, Boston, MA, USA) and Jamovi (Version 2.3.13.0, Sydney, Australia).

### 2.8. Ethical Aspects

The study protocol and its review were approved by an institution review board (Comité de Protection des Personnes Est III) on 4 November 2020 (see Appendix E) and on 3 March 2022 (Appendix F), respectively. This study was conducted in accordance with the principles of the Declaration of Helsinki.

Patients received oral and written information about the nature of the study, its objectives, and expected benefits. Their non-opposition was recorded by one of the senior physicians involved in the research project, in accordance with the French law for observational studies with usual care. If patients were unable to decide for themselves, consent was obtained from their support person.

## 3. Results

### 3.1. General Characteristics

**Figure 1:**
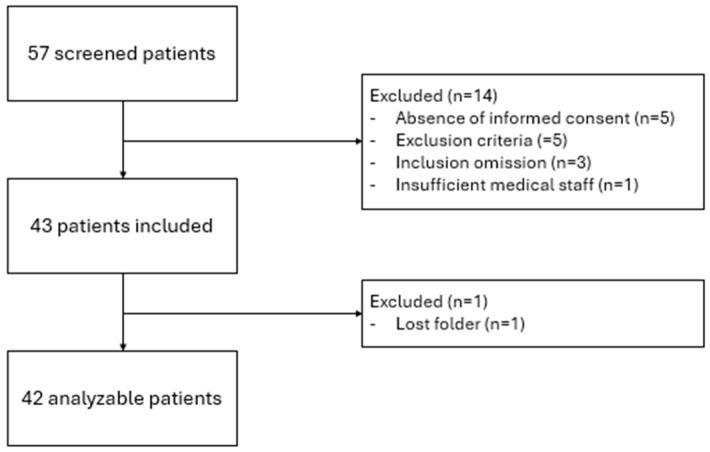
Flow chart.

From 25 May 2021, to 14 July 2022, 57 patients hospitalized for AHF were screened, of which 43 were included in the study. Finally, 42 patients were analyzed due to the loss of one file (Figure 1). No patients were lost for follow-up.

The population was elderly (average age, 85.8 + 8.2 years), mainly male (61.9%), and had multiple comorbidities (Charlson Comorbidity Index (CCI) 8.5 + 2.3). Additionally, 73.8% of patients had a known heart disease and received an average daily dose of 135.5 mg of furosemide at home (range 0–1000 mg) (Table 1).

The Table 2 presents the clinical examination findings, creatinine level, as well as the furosemide posology and number of B-lines at different times during hospitalization.

### 3.2. Reproducibility and Feasibility

After comparing 17 exams conducted by two different physicians, the intraclass correlation coefficient obtained for the total number of B lines was 0.809 (IC 95%: 0.556–0.955) and the Cohen’s kappa coefficient for the presence of pleural effusion was 0.764 (IC 95%: 0.403–1.00).

The 42 patients analyzed accumulated 237 working days of hospitalization. A total of 188 LUS were performed, representing 79.3% of the working days. In other words, 20.7% of the LUS were missing during patient follow-up.

### 3.3. LUS B-Lines, Posology Diuretics, Clinical and Biological Parameters

**Figure 2:**
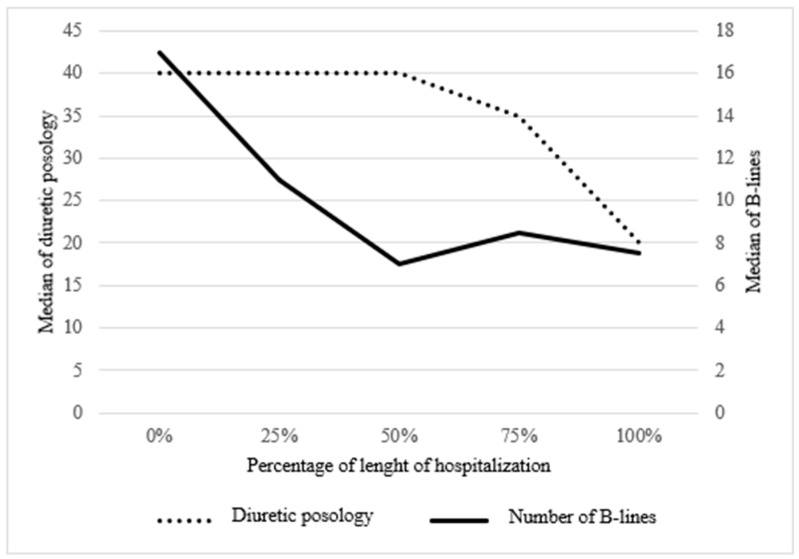
Evolution of median B-lines and median diuretic posology according to time.

The number of B-lines decreased promptly and significantly at the beginning of hospitalization with diuretic therapy, then stabilized in the middle and remained stable until discharge. Diuretic posology remained stable at the beginning of hospitalization, then decreased toward the middle and until discharge (Figure 2 and Table 3).

Crackles also decreased during hospitalization, without any significant relationship to the number of B-lines (Table 4).

At discharge, there was no significant connection between the number of B-lines and the presence or intensity of crackles (Table 5).

**Figure 3:**
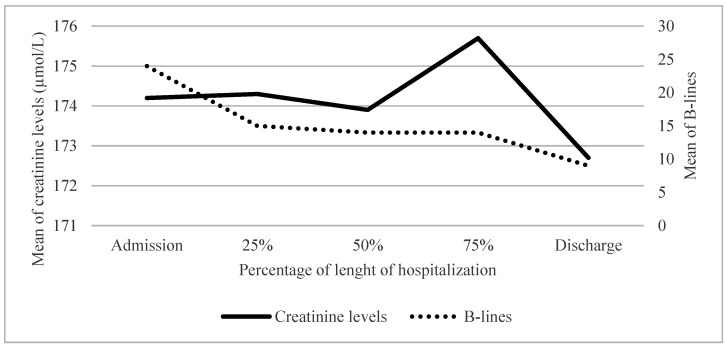
Evolution of number of B-lines and creatinine levels according to time in patients with a history of CKD; CKD = Chronic kidney disease.

**Figure 4:**
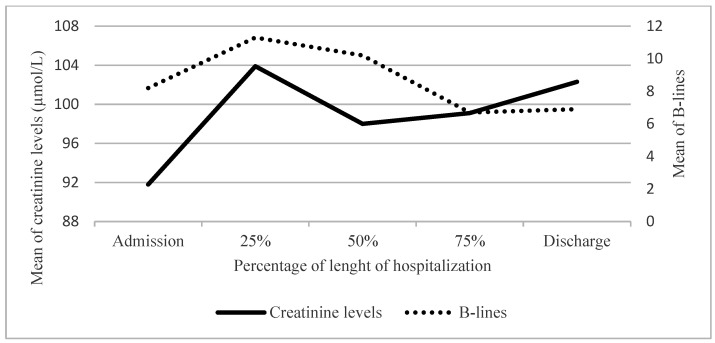
Evolution of number of B-lines and creatinine levels according to time in patients without a history of CKD; CKD = Chronic kidney disease.

Twenty-two patients (52%) had a known history of chronic kidney disease (CKD) with a baseline average creatinine value of 155.7 µmol/L. Their average value was 174.2 µmol/L at hospital admission and remained relatively stable throughout the hospital stay (Figure 3). Among them, 15 (68%) suffered acute kidney injury (AKI). AKI was present in 11 patients (50%) at admission and 4 patients (18%) developed it during hospitalization. The 20 patients (48%) without a history ok CKD had an average creatinine level at admission of 91.8 µmol/L which remained stable throughout their hospital stay (Figure 4). Among them, 7 patients (35%) suffered from AKI with 2 patients (10% having AKI at admission and an additional 5 patients (25%) developed it during hospitalization. Table 6 shows that at admission, patients with a history of CKD had a significantly higher number of B-lines than those without a history of CKD. There was no relationship observed between the evolution of the number of B-lines and creatinine levels.

Within one month of hospitalization, 5 patients (12%) died and 7 patients (20%) were re-hospitalized for AHF. Only one of the deceased patients died after readmission for AHF. The relationship between the number of B-lines and the vital status at 30-days discharge is statistically significant with a *p*-value < 0.001 (Table 7).

## 4. Discussion

In this prospective monocentric study conducted in a post-urgence ward, we examined the relationship among the number of B-lines on LUS, the administrated diuretic posology, and the clinical and biological signs of cardiac overload in hospitalized patients with AHF. We observed an important decrease in the number of B-lines during the first half of hospitalization, followed by a stabilization trend, while the diuretic posology decreased during the second part of hospitalization. At discharge, some of the patients presented subclinical pulmonary overload without crackles but with persistence of B-lines. The presence of B-lines at discharge is significantly associated with a higher risk of mortality at 30-days.

Previous studies examining the relationship between LUS B-lines and diuretic posology in hospitalized patients outside cardiology and intensive care unit are limited. Pang et al. [16] conducted a pilot trial wherein patients consulting for AHF and pulmonary congestion in the ED were divided into two groups to compare standard care and a LUS-guided strategy-of-care in the first six hours. Patients in the intervention group showed a consistent numerical reduction in B-lines during hospitalization, suggesting that protocol driven therapy may facilitate a faster congestion reduction. Panuccio et al. [17] conducted a pilot study in hospitalized patients for AKI in a nephrology ward. They showed that LUS is superior to clinical examination for detecting interstitial lung edema, notably in preclinical stages of congestion. Cardiology groups have also assessed the utility of LUS in adjusting diuretic treatment in outpatient settings. For example, Rivas-Lasarte et al. [18] used LUS to monitor patients after a hospitalization for AHF and showed that patients with LUS-guided adjustment of diuretic treatment had fewer urgent visits and fewer hospitalizations for lower all-cause mortality rates (HR = 0.518 (CI 95%: 0.268–0.998; *p* = 0.049)) compared to those with standard follow-up.

Our results are consistent with previous studies that reported a discrepancy between clinical pulmonary congestion, notably crackles, and LUS B-lines. This discrepancy can occur at both admission and discharge, as some patients may have subclinical pulmonary fluid overload that is not detectable through clinical examination. LUS can identify this clinically silent pulmonary edema. In the study by Platz and al. [7], 35% of patients with acute heart failure did not have crackles at admission, whereas only 6% did not have B-lines. At discharge, Rivas-Lasarte and al. [19] found that 40% of their patients who were “lung dry” to auscultation still had subclinical fluid overload detected by LUS, which seems to have a similarly poor prognosis to overt clinical pulmonary congestion. The poor interobserver agreement of crackles at lung auscultation may also contribute to this discrepancy. For Panuccio et al. [17], in patients with AKI, the agreement between lung crackles and LUS-B lines was poor (κ = 0.02, *p* = 0.63). Ramos-Hernandez et al. [20] compared the interobserver agreement of LUS and lung auscultation, finding good reproducibility for B-lines (κ = 0.81 (CI 95%: 0.81–0.83)) but poor reproducibility for crackles (κ = 0.18 (CI 95%: 0.16–0.20)).

Evaluating lung congestion in patients with AKI is a challenging task. Panuccio et al. [17] showed that in patients with AKI hospitalized in a nephrology ward, hidden lung congestion is more frequent in apparently euvolemic and hypovolemic patients. Approximately 30% of their euvolemic and hypovolemic patients presented clear-cut cardiovascular congestion. According to Ruggenenti et al. [21], patients with AHF and concomitant AKI require urgent and effective therapy, as the combination of both is a stronger predictor of mortality. Those authors therefore emphasized that the priority must be pulmonary decongestion without being limited by altered renal markers. Blair et al. [22] noted that AKI appearing in-hospital reflects aggressive decongestion, as it is frequently associated with reductions in natriuretic peptides, blood pressure, and body weight, which are good prognostic factors. We did not find studies focusing on LUS and the clinical or biological signs of AKI during episodes of AHF. In our study, patients with a history of CKD had more B-lines at admission than those with healthy kidneys. This relationship was significant only at admission. During hospitalization, creatinine levels remained steady in both groups, while the number of B-lines decreased.

The lack of clearance of B-lines at discharge seems to be associated with a high risk of adverse events, such as ED consultation, readmission for AHF, and all-cause mortality. Our results of 30-day all-cause mortality and readmission for AFH are consistent with data from the American Heart Association, which reports rates of 10% and 20–25%, respectively, in patients older than 65 years following an episode of AHF [23]. Cogliati et al. [3], who studied in-patients in an internal medicine department, found that the sonographic score was significantly associated with adverse events at 100 days, with a hazard ratio (HR) of 1.19 (IC 95%: 1.05–1.14; *p* = 0.005). Similarly, E. Platz et al. [24] found a strong relationship between the risk of adverse events and a high number of B-lines at discharge, with an unadjusted HR at 60 days of 3.30 (IC 95% CI: 1.52–7.17; *p* = 0.002). In our study, we found a significant relationship between the number of B-lines and 30-day all-cause mortality. However, we did not find a connection between the number of B-lines and the risk of readmission, which is inconsistent with the literature.

Gargani et al. [4] and Miglioranza et al. [25] concluded that the presence of fewer than 15 B-lines at discharge identifies a subgroup at extremely low risk of adverse events. Using this threshold to determine prognoses, we observed that among our deceased patients, four out of five had ≥15 B-lines, whereas among the surviving patients, only two out of 37 had ≥15 B-lines.

We focused on all-cause mortality and did not collect the cause of death. Several factors may have contributed to this high mortality rate. The average age in the aforementioned studies were 70, 53, and 71 years, respectively. Like us, only Cogliati et al. [3] were interested in elderly patients. The mean age of our patients was 85.6 years (range: 71.7–98.8), and age is a known strong and independent predictor of mortality in patients with HF [26]. Furthermore CCI is an independent predictor of mid-term post-discharge mortality among elderly HF patients [27]. A CCI ≥ 5 is associated with an age-adjusted HR of 77.3 (IC 95% 74.7–79.8) for one-year mortality [28], and our average CCI was 8.5 (range: 5–17). The prognosis seems to be influenced not only by pulmonary edema but also by the triggering factors and underlaying conditions [29,30].

### 4.1. Strenghs

Our study had several strengths. First, we performed eight-zone LUS according to the recommendations in an international consensus statement [15] and interpreted in real time. LUS were performed with patients in the same position, using the same ultrasound device and configuration.

Second, although the extrapolation of our results may be limited by the small sample size and the single center approach, we performed a large number of LUS (188). Two main factors may explain the low number of patients included. First, the COVID-19 pandemic (the department only admitted COVID-19 patients during the months of December 2021 and January 2022, and three COVID-19 clusters slowed down admissions); and second, other departments in our hospital also admitted these patients (the cardiology, geriatrics, and short-stay hospitalization units).

Third, the majority of studies assessing LUS were conducted in cardiology or intensive care units, with few investigations carried out in polyvalent medicine wards. The patients included in our study were predominantly elderly and presented with multiple comorbidities, thus comprising a geriatric population more closely than the younger, less comorbid cohorts typically encountered in cardiology settings. This distinctive patient profile and the characteristics of the department in which it was conducted are part of what makes our study original.

Fourth, practitioners performing the LUS were blinded to the clinical examination. Even though LUS was not performed by the same physician every day, the intraclass correlation coefficient for the number of B-lines was excellent (0,809 (IC 95%: 0.556–0.955)) and comparable to that found in other studies. For instance, Russel et al. [31] reported a coefficient of 0.74 after a trial of approximately 11 exams and two hours of theoretical class. Miglioranza et al. [25] reported a coefficient of 0.98 (95% IC: 0.98–0.99; *p* < 0.0001) in a set of 20 consecutive patients, and M. Mazzola et al. [32] found a coefficient of 0.98 (*p* < 0.0001) in 50 LUS videos evaluated by an expert reader.

Fifth, although doctors were free to prescribe treatment doses based on their assessments, our study positively impacted the implementation of standardized care protocols for AHF treatment in the department.

### 4.2. Limitations

Our study had some limitations. First, the absence of a consensus definition of cardiac congestion and the lack of a gold standard to verify its presence are significant limitations for the diagnosis of AFH. Consequently, physicians relied on a set of clinical, biological, and imaging criteria to establish a diagnosis before including a patient, as recommend by the ESC [12]. There is also no consensus on the definition of ultrasound pulmonary congestion, which is why we decided to count the number of B-lines without setting a specific cut-off. We did not exclude patients with pleural effusion. We decided to record “0” when a pleural effusion prevented the counting of B-lines, which likely led to an underestimation of congestion. This is also one of the limitations of our study.

Second, we did not perform daily LUS on each patient during working days as planned; rather, we completed 79.3% of the intended exams. This can be explained by the high workload and the need for sufficient senior doctors to perform blind tests. We did not find other studies that performed daily LUS, making this an original feature. Gargani et al. [4] performed LUS at admission and discharge with 100% feasibility, while Mazzola et al. [32] conducted LUS at admission, 24 and 48 h after admission, and before discharge. It therefore appears appropriate to adapt the frequency of LUS examinations according to the specific clinical objectives. If the goal is to develop protocols using LUS to guide therapeutic adjustments, performing daily ultrasound assessments would be justified. Conversely, if the objective is to improve long-term prognosis following hospitalization, i.e., by detecting subclinical congestion prior to discharge, then daily LUS examinations would be less relevant and a more targeted approach would be preferable.

Third, during their stay in the ED, our patients received furosemide (an average of 122 mg IV). Some patients stayed for several hours, and we have no ultrasound data from this period. B-lines are dynamic and quickly impacted by diuretic treatment [7]. Consequently, our clinical and ultrasound examination may have shown fewer signs of congestion than when patients first arrived at ED. Pang et al. [16] showed that patients with LUS-guided therapy in the ED had a significantly greater reduction in B-lines in the first 48 h compared to those receiving usual care (*p* = 0.04).

Fourth, the detection of B-lines does not necessarily imply a cardiogenic origin. Other pathologies can also cause of B-lines. None of our patients presented with ARDS, and we excluded patients with fibrosis and pneumonectomy. However, we included patients with infectious pneumonia, which is a common trigger for AHF. We used the total number of B-lines for our analysis, which can be increased in such cases and may constitute a confounding factor. Nevertheless, according to Mazzola et al. [32], LUS can monitor pulmonary decongestion in patients with AHF, whether they have associated pneumonia or not.

Fifth, we conducted the study without considering the left ventricular ejection fraction. Yang et al. [33] did not find significant differences in the B-lines between heart failure with preserved ejection fraction (HFpEF) and reduced ejection fraction (HFrEF) groups. Conversely, Palazzuoli et al. [34] found that patients with HErEF had more B-lines compare to those with HEpEF at admission (*p*-value = 0.04) and at discharge (*p*-value = 0.009). These studies are discordant, indicating a need for further research.

## 5. Conclusions

Over the last decade, pleuropulmonary ultrasound has emerged as an additional tool for diagnosing and monitoring patients who suffer from heart failure.

In this prospective observational study conducted in a multidisciplinary medical ward, we observed a trend toward stagnation in the number of B-lines at the end of hospitalization, while the administered diuretic posology continued to decrease. At the time of discharge, some of our patients presented a subclinical pulmonary overload. Subject to small patient numbers and the limitations describe above, we found a significant relationship between the number of B-lines at discharge and risk of death at 30 days.

In the absence of a gold standard for the quantitative assessment of cardiac overload, LUS is a valuable tool that could help optimize the management of congestion, particularly subclinical congestion, thereby improving the 30-day prognoses. A randomized interventional study is warranted to determine whether a standardized, LUS-guided diuretic titration protocol would lead to better outcomes in AHF patients compared to standard clinical assessment.

## Figures and Tables

**Table 1:** Characteristics of the study population.

	No.	Mean (SD) or No. (%)
Baseline characteristics		
Age, years (extremes)	42	85.8 (SD 8.2) (71.7–98.8)
Male gender	42	26 (61.9%)
BMI (kg/m^2^)	39	26.5 (SD 6.2)
Charlson Comorbidity Index	42	8.5 (SD 2.3)
Chronic kidney disease	42	22 (52%)
Hypertension	42	32 (76%)
Diabetes	42	18 (43%)
Previous cardiac history		

Know heart disease	42	31 (73.8%)
LVEF	25	49.8 (SD 13.5)
Daily per os furosemide posology (mg)	42	132.5 (SD 231.5)
Triggering factor of AHF		
Unknown	42	8 (19.0%)
Anemia	42	9 (21.4%)
Pneumopathy	42	8 (21.4%)
Atrial fibrillation	42	5 (11.9%)
Other bacterial infections (pyelonephritis, erysipelas)	42	4 (9.5%)
Recent decrease in diuretic treatment	42	2 (4.8%)
Non-compliance in healthcare	42	2 (4.8%)
Pulmonary embolism	42	1 (2.4%)
Altitude hypoxemia	42	1 (2.4%)
COVID-19	42	1 (2.4%)
Biological and radiography characteristics at admission		
Hemoglobin (g/dL)	42	11.2 (SD 1.9)
Ferritin (ng/mL)	30	167.1 (SD 199.2)
Transferrin saturation (%)	30	12.4 (SD 9.1)
Creatinine (µmol/L)	42	134.9 (SD 55.0)
Urea (mmol/L)	42	14.7 (SD 9.5)
Natremia (mmol/L)	42	139.8 (SD 3.8)
Kalemia (mmol/L)	41	4.2 (SD 0.7)
NT-pro BNP (ng/L)	41	9041 (SD 8770.6)
Albumin (g/L)	35	32.1 (SD 5.6)
Signs of pulmonary oedema on chest radiography	36	30 (83.3%)
During hospitalization		
Length of hospitalization (days)	42	8.0 (5.1)
Furosemide posology IV received in ED (mg)	42	122.4 (SD 214.5)
Furosemide posology IV/24h on admission (mg)	42	159.2 (SD 256.5)
Intra-venous iron supplementation	42	24 (57.1%)
Dietetic consultation	42	15 (35.7%)
Physiotherapy	42	25 (59.5%)
Therapeutic patient education	42	5 (11.9%)
At discharge		
Daily per os furosemide posology (mg)	42	153.9 (SD 268.5)
Biological check-up at 7 days	42	22 (52.4%)
Consultation with attending physician at 7 days	42	18 (42.9%)
Consultation with cardiologist at 20 days	42	20 (47.6%)

**Table 2:** Evolution of characteristics of patients during hospitalization.

		Admission (0%)	25%	50%	75%	Discharge (100%)
Weight loss Mean (SD) [n]		-	−0.8 (−0.4) [24]	−1.7 (−0.8) [22]	−1.6 (−1.2) [21]	−2.7 (−1.6) [20]
Dyspnea (self-evaluation) Mean (SD) [n]		5.1 (3.2) [34]	3.3 (2.9) [25]	2.1 (2.5) [26]	1.8 (2.3) [25]	1.7 (2.2) [28]
NYHA Mean (SD) [n]		3.1 (1.0) [35]	2.3 (1.0) [27]	2.2 (1.0) [27]	2.1 (0.9) [23]	2.2 (0.9) [27]
Orthopnea % of total [n]	Yes	36.6% [15]	14.7% [5]	9.7% [3]	7.4% [2]	2.6% [1]
	No	63.4% [26]	85.3% [29]	90.3% [28]	92.6% [25]	97.4% [37]
Asthenia (self-evaluation) Mean (SD) [n]		5.6 (3.0) [31]	4.4 (2.7) [23]	3.9 (3.0) [20]	3.3 (3.2) [18]	3.2 (2.6) [21]
Crackles % of total [n]	No	24.4% [10]	32.3% [10]	35.5% [11]	30.0% [9]	44.7% [17]
	Base	26.8% [11]	38.7% [12]	48.4% [15]	53.3% [16]	31.6% [12]
	1/4 lung	12.2% [5]	6.5% [2]	6.5% [2]	10.0% [3]	21.1% [8]
	1/2 lung	29.3% [12]	22.6% [7]	9.7% [3]	6.7% [2]	2.6% [1]
	3/4 lung	7.3% [3]	0.0% [0]	0.0% [0]	0.0% [0]	0.0% [0]
VM abolition % of total [n]	No	61.0% [25]	75.0% [24]	63.3% [19]	63.3% [19]	84.2% [32]
	Base	22.0% [9]	9.4% [3]	23.3% [7]	26.7% [8]	10.5% [4]
	1/4 lung	4.9% [2]	6.2% [2]	3.3% [1]	3.3% [1]	5.3% [2]
	1/2 lung	7.3% [3]	6.2% [2]	6.7% [2]	6.7% [2]	0.0% [0]
	3/4 lung	2.4% [1]	3.1% [1]	3.3% [1]	0.0% [0]	0.0% [0]
	All lung	2.4% [1]	0.0% [0]	0.0% [0]	0.0% [0]	0.0% [0]
Lower limbs oedema % of total [n]	No	33.3% [14]	50.0% [16]	14 (46.7%)	48.3% [14]	56.8% [21]
	Ankle	28.6% [12]	21.9% [7]	12 (40.0%)	44.8% [13]	45.1% [13]
	Knee	28.6% [12]	25.0% [8]	3 (10.0%)	6.9% [2]	5.4% [2]
	Thigh	9.5% [4]	3.1% [1]	1 (3.3%)	0.0% [0]	2.7% [1]
Creatinine (µmol/L) Mean (SD) [n]		133.7 (55.1) [42]	139.1 (51.8) [30]	139.8 (54.0) [26]	144.1 (58.5) [21]	142.8 (57.5) [30]
Furosemide posology (IV, mg) Mean (SD) [n]		162.1 (SD 259.0) [42]	147.4 (224.7) [35]	124.0 (220.2) [36]	80.5 (137.4) [34]	77.0 (134.2) [42]
Pleural effusion % of total [n]	Yes	61.5% [8]	60.0% [15]	48.0% [12]	46.2% [12]	35.7% [10]
	No	38.5% [5]	40.0% [10]	52.0% [13]	53.8% [14]	64.3% [18]
B-lines left Mean (SD) [n]		9.1 (7.5) [13]	6.8 (5.1) [25]	5.6 (5.1) [25]	4.8 (4.1) [26]	4.5 (3.5) [28]
B-lines right Mean (SD) [n]		9.8 (7.1) [13]	8.2 (6.9) [25]	6.3 (4.8) [25]	6.0 (4.2) [26]	3.9 (4.0) [28]
Total B-lines Mean (SD) [n]		18.1 (13.0) [13]	13.4 (10.2) [25]	11.9 (9.4) [25]	10.8 (7.8) [26]	8.4 (6.8) [28]

**Table 3:** Evolution of number of B-lines and diuretic posology according to time.

Hospitalization Time	Median of B-Lines	Median of Diuretic Posology	B-Lines Tendency	Diuretic Posology Tendency	*p*-Value Univariate	*p*-Value multivariate ^1^
0%	17	40			0.941	0.869
25%	11	40	0.65	1	0.901	0.960
50%	7	40	0.64	1	0.216	0.396
75%	8.5	35	1.21	0.88	0.306	0.591
100%	7.5	20	0.88	0.57	0.305	0.737

^1^ Adjustment on age.

**Table 4:** Evolution of number of B-lines according to crackles.

Hospitalization Time	Intensity of Clinic Sign	No.	B-Lines Mean (SD) [Count]	*p* Value ^1^
Admission (0%)	Absent	13	14.7 (9.1) [3]	0.295
	Moderate ^a^		12.8 (14.4) [5]	
	Severe ^b^		25.4(12.3) [5]	
25%	Absent	31	10.3 (11.4) [10]	0.824
	Moderate ^a^		8.7 (9.9) [14]	
	Severe ^b^		11.7 (11.1) [7]	
50%	Absent	31	9.8 (11.8) [11]	0.539
	Moderate ^a^		9.1 (8.3) [17]	
	Severe ^b^		3.0 (3.6) [3]	
75%	Absent	30	12.8 (9.4) [9]	0.223
	Moderate ^a^		7.4 (7.9) [19]	
	Severe ^b^		4.5 (6.4) [2]	
At discharge (100%)	Absent	39	6.6 (7.1) [17]	0.055
	Moderate ^a^		3.9 (6.4) [21]	
	Severe ^b^		20.0 (NA) [1]	

^a^ Crackles in bases and 1/4 lung ^b^ Crackles in 1/2, 3/4 and all lung; ^1^ Linear Model ANOVA.

**Table 5:** Correlation between crackle intensity and the number of B-lines at discharge.

At Discharge	No Crackles	Bases Crackles	¼ Lung Crackles	½ Lung Crackles	¾ Lung Crackles	All Lung Crackles	*p*-Value ^1^
B lines Mean (SD) [n]	6.6 (7.1) [17]	4.1 (7.2) [13]	3.6 (5.4) [8]	20.0 (NA) [1]	0.0 (NA) [0]	0.0 (NA) [0]	0.124

^1^ Linear Model ANOVA.

**Table 6:** B-line evolution according to creatinine measurement.

Hospitalization Time	CDK History	No.	Creatinine in µmol/L Mean (SD) [n]	B-Lines Mean (SD) [n]	*p* Value ^1^
Admission (0%)	Yes	22	174.2 (43.1) [22]	24.2 (11.6) [8]	0.023
	No	20	91.8 (26.9) [20]	8.2 (8.8) [5]	
25%	Yes	22	174.3 (43.8) [15]	14.6 (9.4) [16]	0.450
	No	20	103.9 (31.4) [15]	11.3 (11.7) [9]	
50%	Yes	22	170.5 (49.8) [15]	13.7 (10.2) [12]	0.373
	No	20	98.0 (21.7) [11]	10.2 (8.7) [13]	
75%	Yes	22	171.8 (56.6) [13]	14.4 (8.5) [14]	0.008
	No	20	99.1 (23.3) [8]	6.7 (3.9) [12]	
At discharge (100%)	Yes	22	169.8 (57) [18]	9.2 (7.5) [18]	0.406
	No	20	102.3 (26.9) [12]	6.9 (5.2) [10]	

^1^ Linear Model ANOVA.

**Table 7:** B-lines at discharge according to vital and hospital status 30-days after discharge.

At 30-Days After Discharge		B-Lines Mean (SD) [n] at Discharge	*p*-Value ^1^
Vital status	Death (all causes)	15.2 (10.1) [5]	<0.001
	Alive	3.9 (5.4) [37]	
Hospital status for AHF	Readmission	4.9 (5.4) [8]	0.469
	No readmission	4.1 (5.7) [30]	

^1^ Linear Model ANOVA.

## Data Availability

All documents used are attached to this article. Patient data is not accessible to third parties due to patient data confidentiality.

## Abbreviations

The following abbreviations are used in this manuscript:

AHF	Acute Heart Failure
AKI	Acute Kidney Injury
ARDS	Acute Respiratory Distress Syndrome
BNP	Brain Natriuretic Peptide
CCI	Charlson Comorbidity Index
CKD	Chronic Kidney Disease
ED	Emergency Department
EF	Ejection Fraction
GFR	Glomerular Filtration Rate
HFrEF	Heart Failure Reduced Ejection Fraction
HFpEF	Heart Failure Preserved Ejection Fraction
HR	Hazard Ratio
IV	Intra-venous
LEVF	Left ventricular ejection fraction
LUS	Lung ultrasound
NHYA	New York Heart Association
VM	Vesicular murmur

## Appendix C. Follow-Up Table

**Description of the Data Collected**	**Pre Inclusion** **J0 (Entry Into Service)**	**Inclusion** **T0**	**Daily During Hospitalization**	**Follow-Up Visit** **30 days (+/− 3 Days) After the Last Ultrasound**
Subject Information		✔		
Checking eligibility criteria	✔	✔		
Collection of clinical data		✔	✔	
Collection of biological data		✔	✔ *	
Ultrasound		✔	✔	
Collection of rehospitalizations or vital status				✔
Medical record review				✔

## References

[1] L’état de Santé de la Population en France—Rapport 2017|Direction de la Recherche, des Études, de L’évaluation et des Statistiques. https://drees.solidarites-sante.gouv.fr/publications-documents-de-reference/rapports/letat-de-sante-de-la-population-en-france-rapport-2017.

[2] Coiro S., Rossignol P., Ambrosio G., Carluccio E., Alunni G., Murrone A., Tritto I., Zannad F., Girerd N. (2015). Prognostic value of residual pulmonary congestion at discharge assessed by lung ultrasound imaging in heart failure. Eur. J. Heart Fail..

[3] Cogliati C., Casazza G., Ceriani E., Torzillo D., Furlotti S., Bossi I., Vago T., Costantino G., Montano N. (2016). Lung ultrasound and short-term prognosis in heart failure patients. Int. J. Cardiol..

[4] Gargani L., Pang P.S., Frassi F., Miglioranza M.H., Dini F.L., Landi P., Picano E. (2015). Persistent pulmonary congestion before discharge predicts rehospitalization in heart failure: A lung ultrasound study. Cardiovasc. Ultrasound.

[5] Torino C., Gargani L., Sicari R., Letachowicz K., Ekart R., Fliser D., Covic A., Siamopoulos K., Stavroulopoulos A., Massy Z.A. (2016). The Agreement between Auscultation and Lung Ultrasound in Hemodialysis Patients: The LUST Study. Clin. J. Am. Soc. Nephrol. CJASN.

[6] Riocreux C.A.M. (2019). Thèse de Médecine: Optimisation des Diurétiques Dans la Décompensation de L’insuffisance Cardiaque Chronique: Rationnel e Tdescription de L’étude ProDUCT-HF. Ph.D. Thesis.

[7] Platz E., Merz A.A., Jhund P.S., Vazir A., Campbell R., McMurray J.J. (2017). Dynamic changes and prognostic value of pulmonary congestion by lung ultrasound in acute and chronic heart failure: A systematic review. Eur. J. Heart Fail..

[8] Girerd N., Seronde M.-F., Coiro S., Chouihed T., Bilbault P., Braun F., Kenizou D., Maillier B., Nazeyrollas P., Roul G. (2018). Integrative Assessment of Congestion in Heart Failure Throughout the Patient Journey. JACC Heart Fail..

[9] Martindale J.L., Wakai A., Collins S.P., Levy P.D., Diercks D., Hiestand B.C., Fermann G.J., deSouza I., Sinert R. (2016). Diagnosing Acute Heart Failure in the Emergency Department: A Systematic Review and Meta-analysis. Acad. Emerg. Med. Off. J. Soc. Acad. Emerg. Med..

[10] Lichtenstein D.A., Mezière G.A. (2008). Relevance of lung ultrasound in the diagnosis of acute respiratory failure: The BLUE protocol. Chest.

[11] Öhman J., Harjola V.-P., Karjalainen P., Lassus J. (2018). Focused echocardiography and lung ultrasound protocol for guiding treatment in acute heart failure. ESC Heart Fail..

[12] Oxford Academic (2021). 2021 ESC Guidelines for the Diagnosis and Treatment of Acute and Chronic Heart Failure. Eur. Heart J..

[13] Frasure S.E., Matilsky D.K., Siadecki S.D., Platz E., Saul T., Lewiss R.E. (2015). Impact of patient positioning on lung ultrasound findings in acute heart failure. Eur. Heart J. Acute Cardiovasc. Care.

[14] Buessler A., Chouihed T., Duarte K., Bassand A., Huot-Marchand M., Gottwalles Y., Pénine A., André E., Nace L., Jaeger D. (2020). Accuracy of Several Lung Ultrasound Methods for the Diagnosis of Acute Heart Failure in the ED: A Multicenter Prospective Study. Chest.

[15] Platz E., Jhund P.S., Girerd N., Pivetta E., McMurray J.J.V., Peacock W.F., Masip J., Martin-Sanchez F.J., Miró Ò., Price S. (2019). Expert consensus document: Reporting checklist for quantification of pulmonary congestion by lung ultrasound in heart failure. Eur. J. Heart Fail..

[16] Pang P.S., Russell F.M., Ehrman R., Ferre R., Gargani L., Levy P.D., Noble V., Lane K.A., Li X., Collins S.P. (2021). Lung Ultrasound-Guided Emergency Department Management of Acute Heart Failure (BLUSHED-AHF): A Randomized Controlled Pilot Trial. JACC Heart Fail..

[17] Panuccio V., Tripepi R., Parlongo G., Mafrica A., Caridi G., Catalano F., Marino F., Tripepi G., Mallamaci F., Zoccali C. (2020). Lung ultrasound to detect and monitor pulmonary congestion in patients with acute kidney injury in nephrology wards: A pilot study. J. Nephrol..

[18] Rivas-Lasarte M., Álvarez-García J., Fernández-Martínez J., Maestro A., López-López L., Solé-González E., Pirla M.J., Mesado N., Mirabet S., Fluvià P. (2019). Lung ultrasound-guided treatment in ambulatory patients with heart failure: A randomized controlled clinical trial (LUS-HF study). Eur. J. Heart Fail..

[19] Rivas-Lasarte M., Maestro A., Fernández-Martínez J., López-López L., Solé-González E., Vives-Borrás M., Montero S., Mesado N., Pirla M.J., Mirabet S. (2020). Prevalence and prognostic impact of subclinical pulmonary congestion at discharge in patients with acute heart failure. ESC Heart Fail..

[20] Ramos-Hernández C., Botana-Rial M., Núñez-Fernández M., Lojo-Rodríguez I., Mouronte-Roibas C., Salgado-Barreira Á., Ruano-Raviña A., Fernández-Villar A. (2021). Validity of Lung Ultrasound: Is an Image Worth More Than a Thousand Sounds?. J. Clin. Med..

[21] Ruggenenti P., Remuzzi G. (2011). Worsening kidney function in decompensated heart failure: Treat the heart, don’t mind the kidney. Eur. Heart J..

[22] Blair J.E.A., Pang P.S., Schrier R.W., Metra M., Traver B., Cook T., Campia U., Ambrosy A., Burnett J.C., Grinfeld L. (2011). Changes in renal function during hospitalization and soon after discharge in patients admitted for worsening heart failure in the placebo group of the EVEREST trial. Eur. Heart J..

[23] Virani S.S., Alonso A., Aparicio H.J., Benjamin E.J., Bittencourt M.S., Callaway C.W., Carson A.P., Chamberlain A.M., Cheng S., Delling F.N. (2021). Heart Disease and Stroke Statistics—2021 Update. Circulation.

[24] Platz E., Campbell R.T., Claggett B., Lewis E.F., Groarke J.D., Docherty K.F., Lee M.M.Y., Merz A.A., Silverman M., Swamy V. (2019). Lung Ultrasound in Acute Heart Failure: Prevalence of Pulmonary Congestion and Short- and Long-Term Outcomes. JACC Heart Fail..

[25] Miglioranza M.H., Gargani L., Sant’Anna R.T., Rover M.M., Martins V.M., Mantovani A., Weber C., Moraes M.A., Feldman C.J., Kalil R.A.K. (2013). Lung ultrasound for the evaluation of pulmonary congestion in outpatients: A comparison with clinical assessment, natriuretic peptides, and echocardiography. JACC Cardiovasc. Imaging.

[26] Komajda M., Hanon O., Hochadel M., Lopez-Sendon J.L., Follath F., Ponikowski P., Harjola V.-P., Drexler H., Dickstein K., Tavazzi L. (2009). Contemporary management of octogenarians hospitalized for heart failure in Europe: Euro Heart Failure Survey II. Eur. Heart J..

[27] Formiga F., Moreno-Gonzalez R., Chivite D., Franco J., Montero A., Corbella X. (2018). High comorbidity, measured by the Charlson Comorbidity Index, associates with higher 1-year mortality risks in elderly patients experiencing a first acute heart failure hospitalization. Aging Clin. Exp. Res..

[28] Bannay A., Chaignot C., Blotière P.-O., Basson M., Weill A., Ricordeau P., Alla F. (2016). The Best Use of the Charlson Comorbidity Index with Electronic Health Care Database to Predict Mortality. Med. Care.

[29] Freund Y., Cachanado M., Delannoy Q., Laribi S., Yordanov Y., Gorlicki J., Chouihed T., Féral-Pierssens A.-L., Truchot J., Desmettre T. (2020). Effect of an Emergency Department Care Bundle on 30-Day Hospital Discharge and Survival Among Elderly Patients with Acute Heart Failure: The ELISABETH Randomized Clinical Trial. JAMA.

[30] Teixeira A., Parenica J., Park J.J., Ishihara S., AlHabib K.F., Laribi S., Maggioni A., Miró Ò., Sato N., Kajimoto K. (2015). Clinical presentation and outcome by age categories in acute heart failure: Results from an international observational cohort. Eur. J. Heart Fail..

[31] Russell F.M., Ferre R., Ehrman R.R., Noble V., Gargani L., Collins S.P., Levy P.D., Fabre K.L., Eckert G.J., Pang P.S. (2020). What are the minimum requirements to establish proficiency in lung ultrasound training for quantifying B-lines?. ESC Heart Fail..

[32] Mazzola M., Pugliese N.R., Zavagli M., De Biase N., Bandini G., Barbarisi G., D’Angelo G., Sollazzo M., Piazzai C., David S. (2021). Diagnostic and Prognostic Value of Lung Ultrasound B-Lines in Acute Heart Failure with Concomitant Pneumonia. Front. Cardiovasc. Med..

[33] Yang F., Wang Q., Zhi G., Zhang L., Huang D., Shen D., Zhang M. (2017). The application of lung ultrasound in acute decompensated heart failure in heart failure with preserved and reduced ejection fraction. Echocardiography.

[34] Palazzuoli A., Ruocco G., Beltrami M., Nuti R., Cleland J.G. (2018). Combined use of lung ultrasound, B-type natriuretic peptide, and echocardiography for outcome prediction in patients with acute HFrEF and HFpEF. Clin. Res. Cardiol. Off. J. Ger. Card. Soc..

